# Cardiac tamponade in a cat with pericardial mesothelioma

**DOI:** 10.1007/s11259-026-11172-w

**Published:** 2026-03-28

**Authors:** Fabio Spina, Margherita Orlandi, Raffaele Preziosi, Giuseppe Giglia, Monica Sforna, Francesco Birettoni

**Affiliations:** 1 Freelance, Rome, Italy; 2 MyLav Veterinary Diagnostic Laboratory, Milan, Italy; 3 Ambulatorio Veterinario Pacchiarotti, Rome, Italy; 4 Department of Veterinary Medicine, University of Perugia, Perugia, Italy

**Keywords:** Pericardial mesothelioma, Heart tumors, Cardiac tamponade, Mesothelioma immunohistochemistry, Cat

## Abstract

Pericardial effusion is uncommon in cats and most often secondary to congestive heart failure, feline infectious peritonitis or neoplasia. Primary cardiac tumors are rare in companion animals, with their occurrence being particularly uncommon in cats and among these, mesothelioma is exceptionally rare. Its antemortem diagnosis presents a considerable challenge due to the often-mild nature of intrapericardial fluid accumulation and the difficulty in cytologically differentiating neoplastic cells from reactive mesothelial cells. A 12-year-old Domestic Shorthair cat was presented for dyspnea and weakness. Ultrasound examination revealed concurrent pleural, peritoneal and pericardial effusion. Pericardial effusion appeared densely particulate with multiple small nodules on the epicardial surface. Pericardiocentesis acutely resolved the cardiac tamponade, but recurrence prompted subtotal pericardiectomy. Histopathology of surgical biopsies suggested pericardial mesothelioma. Despite surgery, the cat’s clinical condition deteriorated, leading to euthanasia one month later. Autopsy, histopathology and immunohistochemistry (positive for cytokeratin AE1/AE3, vimentin, calretinin and HBME-1) confirmed pericardial mesothelioma with pleural dissemination. This report describes the clinical, diagnostic, and pathological features of a rare case of feline pericardial mesothelioma and highlights the diagnostic value of a multimodal approach.

## Background

Pericardial effusion is an uncommon pathologic condition in cats and is typically secondary to underlying diseases such as congestive heart failure (CHF), feline infectious peritonitis (FIP), or neoplasia (Rush et al. 1990; Davidson et al. 2008; Hall et al. 2007). In a large retrospective study of 146 cats with echocardiographically confirmed pericardial effusion, CHF was the most frequent cause (approximately 75%), while neoplasia accounted for a smaller proportion (Hall et al. 2007). Primary cardiac tumors are rare in cats and infrequently cause significant pericardial effusion (Aupperle et al. 2007); among neoplastic causes, lymphoma proved to be the most reported cardiac neoplasm (Zoia et al. 2004; Amati et al. 2014; Treggiari et al. 2015). In contrast to dogs, where neoplastic pericardial effusions often lead to cardiac tamponade (with masses detectable in up to 50% of cases in some series), cardiac tamponade secondary to neoplastic effusion is exceptional in cats (Hall et al. 2007; Johnson et al. 2004).

Pericardial mesothelioma is an extremely rare malignant neoplasm in cats, typically presenting with hemorrhagic or serosanguineous pericardial effusion and often without a grossly or echocardiographically detectable mass (Kienle 1998). Recently, mesotheliomas have also been described in large felids, with primary or secondary pericardial involvement reported in 8 of the 10 cases (Sheldon et al. 2025). Antemortem diagnosis is particularly challenging due to the cytologic difficulty in distinguishing neoplastic mesothelial cells from reactive mesothelium, as well as the nonspecific gross appearance of the pericardium. To date, most reported cases of feline mesothelioma involve the pleura or peritoneum, with only a handful of pericardial (or pleuro-pericardial) mesotheliomas documented in the veterinary literature (Gabriel Filho et al. 2015; Davidson et al. 2008; Hall et al. 2007; Tilley et al. 1975; Gourlay et al. 2007; Schlueter et al. 2021).

This report describes the clinical presentation, diagnostic approach (including echocardiography and surgical findings), palliative surgical management (pericardiectomy), and definitive postmortem diagnosis of primary pericardial mesothelioma causing massive pericardial effusion and cardiac tamponade in a cat.

## Case report

A 12-year old, neutered male Domestic Shorthair (DSH) cat was referred for evaluation of acute weakness, anorexia and dyspnea. The cat had been boarded in a cattery two weeks prior. Physical examination revealed depression, tachypnea (45 breaths/min), weak femoral pulses (heart rate 180 bpm), mild dehydration (approximately 5%), moderate abdominal distension, body condition score 4/9, pale mucous membranes, and prolonged capillary refill time (3 s). On auscultation heart sounds were muffled in accordance with cardiogenic or obstructive shock.

**Fig. 1:**
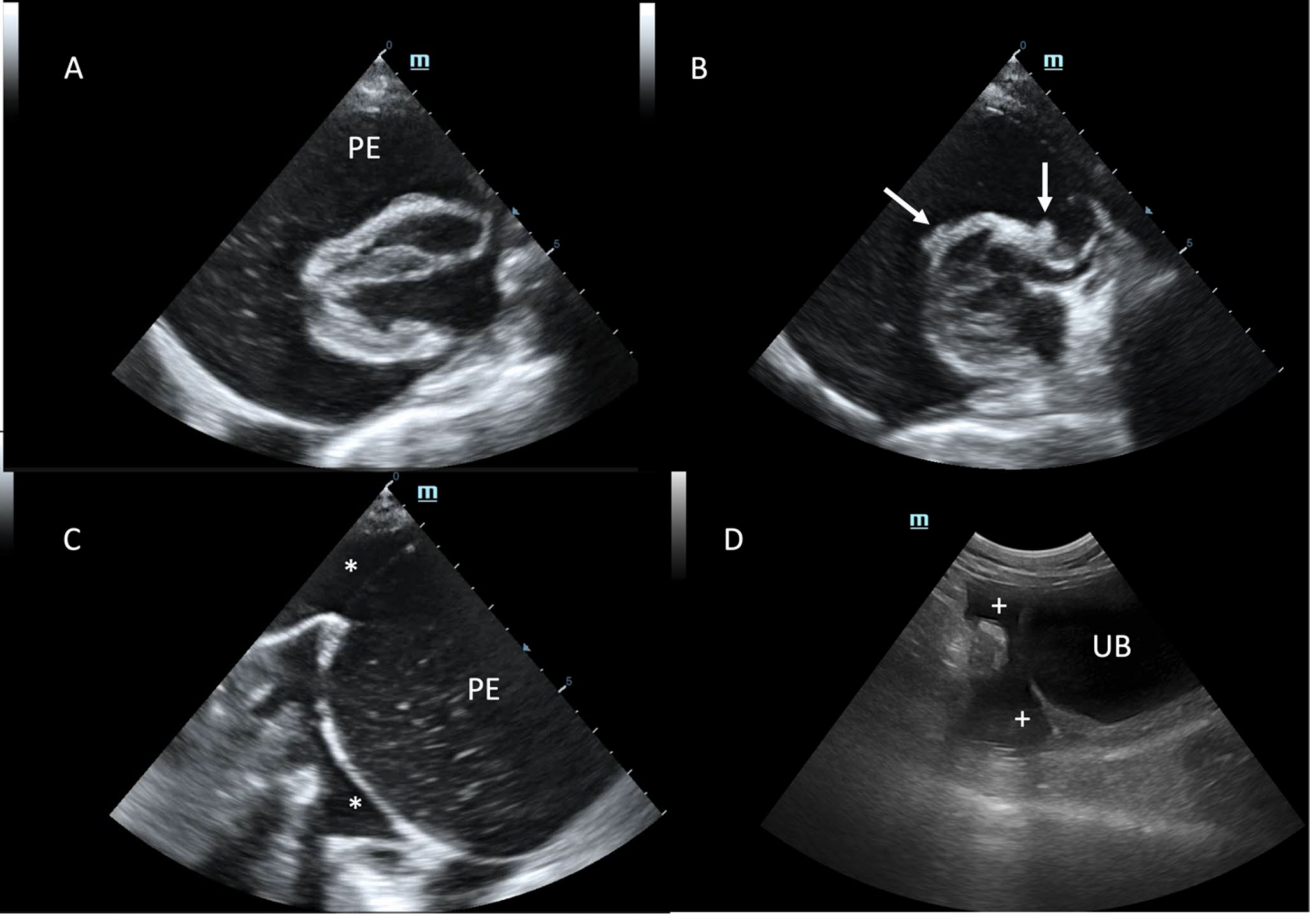
**A** - severe pericardial effusion (PE) causing diastolic collapse of the right heart; **B** – small sessile nodules protrude from the epicardial surface (arrows); C – the pericardial fluid (PE) appeared roughly and densely corpuscolated while the pleural fluid (asteriscs) completely anechoic; **C** – even peritoneal effusion (plus signs) was anechoic and mostly collected cranial to the urinary bladder (UB)

Thoracic radiographs showed mild pleural effusion obscuring the ventral cardiac silhouette. Echocardiography demonstrated severe, densely particulate intrapericardial fluid associated with diastolic collapse of the right atrium and ventricle, confirming cardiac tamponade (Fig. 1).

The epicardium was diffusely thickened and irregular, with numerous small sessile nodules. Trivial tricuspid insufficiency was also noted. Pleural and abdominal effusions were also present in mild to moderate amount and both appeared anechoic.

Under ultrasound guidance, 130 mL of whitish pleural fluid, 100 mL of turbid pericardial fluid, and 120 mL of slightly pink serosanguineous peritoneal fluid were aspirated and submitted to an external laboratory for analysis. Post-drainage, respiratory and cardiac rate normalized (25 breathe per minute and 130 beats per minute, respectively). Hematological and biochemical analyses revealed no abnormalities and serology for FIV, FeLV, and heartworm was negative.

Fluid analysis classified the pericardial effusion as a high protein modified transudate (protein 4.1 g/dL), the pleural effusion as chylous, and the peritoneal effusion as a transudate. Cytology of pericardial and pleural fluids showed reactive mesothelial cells without atypia, rare neutrophils, lymphocytes, and foamy macrophages.

The patient remained stable for the next 12 h after the drainage and was discharged with a prescription of Amoxicillin/Clavulanic acid (Synulox^®^, Pfizer) 12.5 mg/kg BID and Prednisolone (Prednicortone^®^, Dechra) 0.5 mg/kg BID.

Despite the therapy, pericardial effusion reoccurred, and the patient was drained after 10 and 20 days after the first presentation. However, the cat remained free from pleural and peritoneal effusion. To manage the effusion and to collect a pericardial biopsy, subtotal pericardiectomy was proposed and accepted by the owner. The cat was premedicated with Dexmedetomidine (Dexdomitor^®^, Vetoquinol) 0,0125 mg/kg and Metadone (Semfortan^®^, Dechra) 0,25 mg/kg. Anesthesia was inducted with Propofol (Propovet^®^, Zoetis) 2,5 mg/kg and maintained with isoflurane (Isofluran Vet^®^, Boheringer). Lidocaine (Lidocaina^®^ 2%, Ecuphar) was used for local subcutaneous anesthesia from the third to fifth intercostal space of the right hemithorax. The skin incision was performed at the fourth intercostal space and the pericardium was partially removed as described in bibliography (Fossum et al. 2004). The pericardium was thick and the presence of numerous nodules, both pericardial and epicardial, was confirmed. No apparent macroscopic alterations of the pleural surface were noted. After partial removal of the pericardium a thoracic drainage was introduced, and the thoracic cavity was routinely closed.

After three days the thoracic drainage was removed because minimal presence of fluid was aspirated, and the cat was sent home with an oral therapy consisting of Enrofloxacin (Xeden^®^, Ceva) 5 mg/kg/die for 7 days and Meloxicam (Meloxidyl^®^, Ceva) 0,05 mg/kg/die for 3 days.

**Fig. 2:**
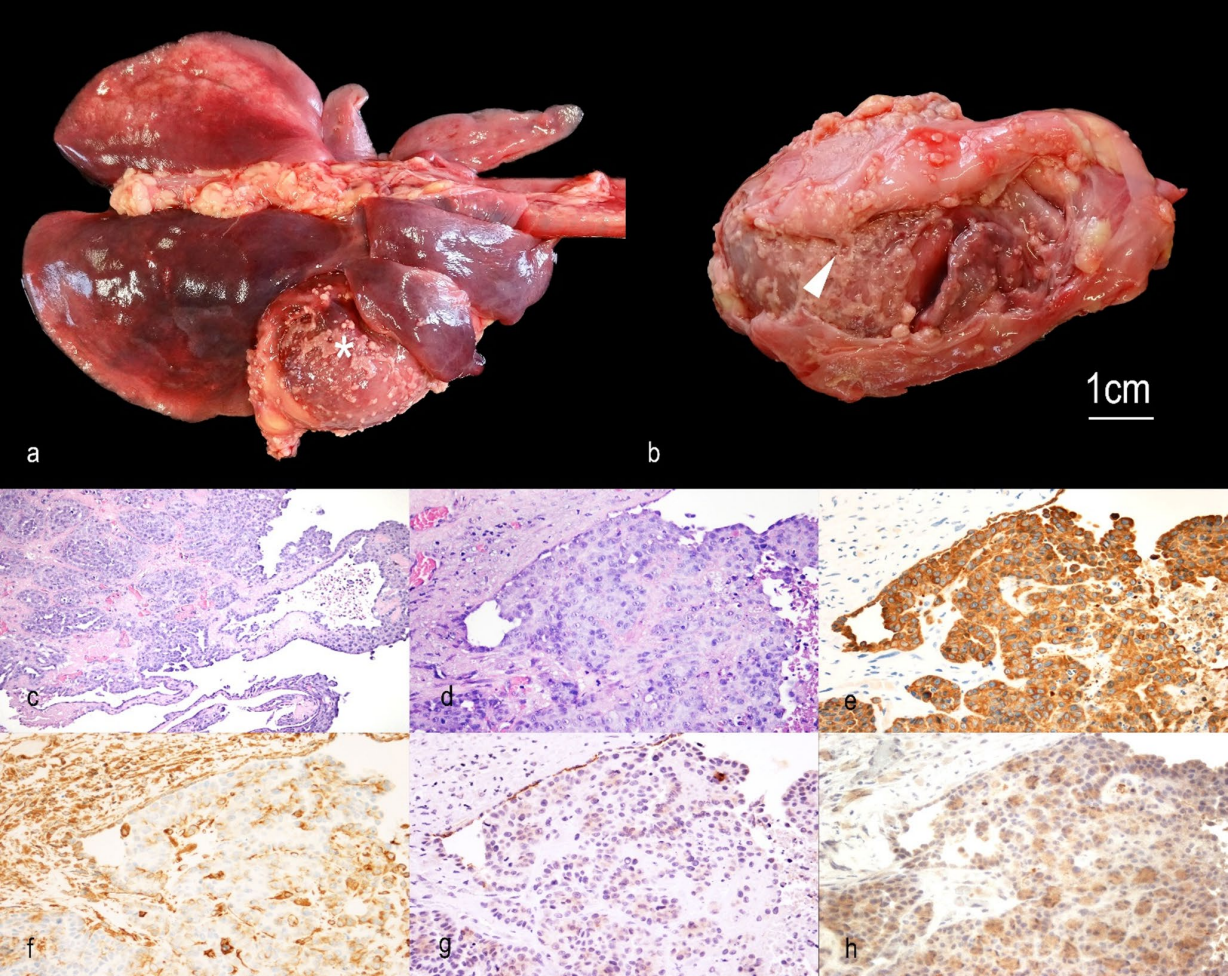
**Macroscopic (a-b)**,** histologic (c-d) and immunohistochemical features (e-h) of pericardial mesothelioma in a cat. (a)** Macroscopically, the pericardium shows externally, numerous, multifocal to coalescing, firm, yellow to gray nodules and plaques (asterisk); **(b)** The pericardium appears diffusely thickened, with an irregular yellow-brown surface, this appears multifocally fused with the epicardium (arrowhead), which shows similar nodules and plaques; **(c)** Nodular to plaque-like pericardial and epicardial formations are composed of solid areas, tubular-like or papillary structures (Hematoxylin and Eosin, x200); **(d)** Polygonal neoplastic cells show moderate eosinophilic cytoplasm and round to oval nuclei with vesicular chromatin and multiple prominent nucleoli (Hematoxylin and Eosin, x400); **(e)** Neoplastic cells and the adjacent normal mesothelium show strong and diffuse positivity to cytokeratin (Anti-Cytokeratin AE1/AE3 IHC, x400); **(f)** Neoplastic cells and the adjacent normal mesothelium show strong and diffuse positivity to vimentin (Anti-Vimentin IHC, x400); **(g)** Neoplastic cells and the adjacent normal mesothelium show moderate and diffuse positivity to HBME-1 (Anti-HBME-1 IHC, x400); **(h)** Neoplastic cells and the adjacent normal mesothelium show moderate and diffuse positivity to calretinin (Anti-Calretinin IHC, x400)

Surgical biopsy of the pericardium was performed and submitted to a private diagnostic laboratory (MyLav, Italy), fixed in 10% neutral-buffered formalin, processed, and embedded in paraffin wax. Three-micrometer sections were cut and stained with hematoxylin and eosin (H&E). Microscopically, there were portions of pericardium surrounded by fibroadipose tissue and involved by a densely cellular, unencapsulated neoplasm. This was composed by polygonal cells arranged in lobules and tubulo-papillary projections, supported by scant fibrovascular stroma and with an evident contiguity with the mesothelial lining. Neoplastic cells had defined margins, moderate amounts of eosinophilic cytoplasm, a round central nucleus with irregularly dispersed chromatin, and a prominent central single nucleolus. Anisocytosis and anisokaryosis were marked, and the mitotic count was 5 per 2.37 mm² with atypical mitotic figures (2 in 2.37 mm²). The neoplastic tissue was affected by small foci of intratumoral necrosis, and the stroma was infiltrated by aggregates of lymphocytes, plasma cells, and hemosiderophages.

As the owners refused chemotherapy, supportive care was started. After the surgery the cat recovered well but the general conditions declined progressively and one month later the cat was euthanized.

The animal was presented for autopsy at the Veterinary Pathology Diagnostic Service, Department of Veterinary Medicine, University of Perugia, Italy. The state of preservation was adequate, and the nutritional condition was adequate. A reddish, frothy fluid discharge was observed from the nostrils. A moderate amount of pleural sero-hemorrhagic effusion (approximately 15 mL) mixed with fibrin clots was identified in the thorax. Numerous firm yellow nodules (~ 0.3 cm in diameter) were scattered on the parietal pleura, particularly on the diaphragm and rib cage. The pericardium appeared diffusely thickened, with an irregular yellow-brown surface, and was multifocally fused with the epicardium. The epicardium displayed disseminated nodular to plaque-like formations on both the atrial and ventricular surfaces. A few small nodules were also present on the pleura of the right middle lung lobe. In the abdominal cavity, a sero-hemorrhagic effusion of approximately 5 mL was present. No macroscopic alterations or lesions were observed in the abdominal organs. To further characterize the suspected neoplastic process, samples were collected for histopathological and immunohistochemical analysis. Following standard procedures, formalin-fixed, paraffin-embedded samples were sectioned at 3 μm and stained with hematoxylin and eosin (H&E). Additional sections were placed on poly-L-lysine-coated slides, dewaxed, and rehydrated for immunohistochemical analysis. Immunolabeling was performed using anti-cytokerain (AE1/AE3), vimentin, Hector Battifora mesothelial-1 (HBME-1) and calretinin antibodies (Table 1), followed by a streptavidin–biotin–horseradish peroxidase detection system (Abcam, Cambridge, UK). To visualize the immunolabeling of selected antigens, 3-amino-9-ethylcarbazole (Abcam, Cambridge, UK) was applied. Positive and negative controls (omitting the primary antibody) were included to ensure technical validity. On histology, multiple sections of cardiac and pericardial tissue were examined, revealing multifocal areas of epi- and pericardial fusion. These areas were characterized by a moderately cellular proliferation of polygonal cells forming papillary structures, plaques, and rare tubular-like structures. The cells exhibited a moderate nucleus-to-cytoplasm ratio and moderate eosinophilic cytoplasm. The nuclei appeared round to oval with vesicular chromatin and multiple prominent nucleoli. Anisocytosis and anisokaryosis were occasionally marked, with a mitotic count of 3 per 2.37 mm². The neoplastic cells were embedded within abundant mature fibrous connective tissue, with mild and scattered multifocal lymphocytic infiltrates. Small areas of coagulative necrosis and fibrin exudation were also observed. Histopathologic criteria were considered compatible with an epithelioid mesothelioma. On immunohistochemistry, cytokeratin (AE1/AE3) and HBME-1 were expressed in the cytoplasm of 90% of neoplastic cells, with moderate diffuse positivity. HBME-1 expression was stronger in normal mesothelium compared to neoplastic mesothelial cells. Calretinin was expressed in the cytoplasm of 85% of neoplastic cells, showing mild to moderate diffuse positivity, with multifocal areas of strong, granular staining. Vimentin was expressed in 60% of neoplastic cells and diffusely in stromal cells (Fig. 2).

**Table 1:** Immunohistochemical markers details performed to characterize the mesothelial proliferation

Marker	Antibody Clonality	Retrieval	Incubation	Manufacturer
Cytokeratin	Mouse monoclonal (AE1/AE3)	ph 9, TRIS-EDTA	1:200; 2 h RT	Dako
Vimentin	Mouse monoclonal (clone V9)	ph 9, TRIS-EDTA	1:250; 2 h RT	Dako
Calretinin	Mouse monoclonal (calretinin H-5)	ph 9, TRIS-EDTA	1:50; 2 h RT	Santa Cruz Biotecnology
HBME-1	Mouse monoclonal (HBME-1)	ph 6, TRIS-EDTA	RTU; 16 min RT	Cell Marque

HBME-1: Hector Battifora mesothelial-1.

RTU: ready to use.

## Discussion

Pericardial effusion in cats is a condition most commonly associated with congestive heart failure (CHF) and feline infectious peritonitis (FIP), and only rarely with neoplasia. In neoplastic cases, an echocardiographically detectable mass is identified in only around 5% of instances, in contrast to dogs, where this percentage reaches up to 50% in some studies (Hall et al. 2007; Johnson et al. 2004; MacDonald et al. 2009).

Although pericardial feline mesothelioma has been already described, to our knowledge, this is the first reported case of pericardial mesothelioma inducing massive pericardial effusion and cardiac tamponade in a cat, with a clinical presentation simultaneously characterized by low-output (anterograde) and right-sided congestive heart failure. Rather than being directly related to the neoplasia, we considered the pleural and peritoneal effusions to be secondary to the cardiac tamponade, as they did not recur after resolution of the tamponade.

Ultrasound proved to be a valuable tool for detecting and characterizing cavitary effusions. In this particular case, it not only identified fluid in all three mesothelial cavities but also characterized the pericardial fluid as grossly particulate (in contrast to the pleural and peritoneal fluids) and allowed detection of multiple small, irregular nodules on the epicardial surface. Visually, the ultrasound images and gross anatomic findings showed a strong correlation, highlighting what appears to be a peculiar feature of mesothelioma.

Based on the histological features, an epithelioid mesothelioma with a tubulopapillary pattern was diagnosed. The epithelioid subtype appears to be the most prevalent in both human and cats (Husain et al. 2024; Bacci et al. 2006). In this case the main differential diagnosis to consider was a metastatic adenocarcinoma (carcinomatosis) and a diffuse reactive mesothelial proliferation; immunohistochemical analysis, in association with histological and gross findings, played a crucial role in confirming the diagnosis of mesothelioma in this feline case. In the selected panel each marker provided a specific diagnostic contribution. This approach was in line with the diagnostic recommendations for the pathologic diagnosis of human mesotheliomas (Husain et al. 2024). The expression of broad-spectrum cytokeratin (AE1/AE3) and calretinin are recognized and recommended as reliable parameters of mesothelial origin in human medicine. Calretinin, a calcium-binding protein, is consistently expressed in mesothelial cells in humans, while in cats no previous positivity has been reported in mesothelioma (Bacci et al. 2006). The co-expression of these two markers provided in the current case solid evidence supporting the mesothelial origin of the proliferation. HBME-1 is another marker commonly expressed in human mesotheliomas, and its use has also been reported in feline mesothelioma cases (Bacci et al. 2006); interpreted in combination with other mesothelial markers, as in the current case, strengthens the diagnostic confidence of the diagnosis. Although not specific for the mesothelial origin, vimentin, a marker of mesenchymal origin, widely expressed in mesotheliomas, was also included to assess the basic immunohistochemical reactivity of the tissue as recommended from the human pathology guidelines.

Our findings suggest that primary pericardial mesothelioma should be considered in the differential diagnosis for cats presenting with massive pericardial effusion and cardiac tamponade, particularly when echocardiography reveals densely particulate fluid and epicardial irregularity. Although the prognosis remains poor due to the aggressive nature of the disease, in this case pericardiectomy provided temporary palliation, and the biopsies obtained during the procedure were crucial for histopathological and immunohistochemical confirmation of the definitive diagnosis.

## Data Availability

No datasets were generated or analysed during the current study.
